# Crystal structure of methyl 2-hy­droxy-5-[(4-oxo-4,5-di­hydro-1,3-thia­zol-2-yl)amino]benzoate

**DOI:** 10.1107/S2056989015006416

**Published:** 2015-04-09

**Authors:** Shaaban K. Mohamed, Joel T. Mague, Mehmet Akkurt, Hajjaj H. M. Abdu-Allah, Mustafa R. Albayati

**Affiliations:** a Chemistry and Environmental Division, Manchester Metropolitan University, Manchester M1 5GD, England; b Chemistry Department, Faculty of Science, Minia University, 61519 El-Minia, Egypt; c Department of Chemistry, Tulane University, New Orleans, LA 70118, USA; d Department of Physics, Faculty of Sciences, Erciyes University, 38039 Kayseri, Turkey; e Department of Pharmaceutical Organic Chemistry, Faculty of Pharmacy, Assiut University, 71515 Assiut, Egypt; f Kirkuk University, College of Science, Department of Chemistry, Kirkuk, Iraq

**Keywords:** crystal structure, amino­salicylic acid, thia­zolidinones, hydrogen bonding

## Abstract

The title compound, C_11_H_10_N_2_O_4_S, crystallized with two independent mol­ecules (*A* and *B*) in the asymmetric unit. They differ primarily in the rotational orientation of the five-membered heterocyclic ring. In mol­ecule *A* this ring is inclined to the benzene ring by 48.17 (8)°, while in mol­ecule *B* the same dihedral angle is 23.07 (8)°. In each mol­ecule there is an intra­molecular O—H⋯O hydrogen bond involving the adjacent hydroxyl group and the ester carbonyl O atom. In the crystal, the *A* mol­ecules are linked *via* pairs of N—H⋯N hydrogen bonds, forming inversion dimers. These dimers are linked to the *B* mol­ecules *via* N—H.·O, C—H⋯O and C—H⋯S hydrogen bonds forming corrugated sheets lying parallel to (102).

## Related literature   

For pharmaceutical and chemotherapeutic properties of amino salicylic acid derivatives, see: Abdel-Alim *et al.* (2005 ▸); Abdu-Allah *et al.* (2005 ▸); Koelink *et al.* (2010 ▸). For general biological activities of thia­zolidinone scaffold compounds, see: Tripathi *et al.* (2014 ▸).
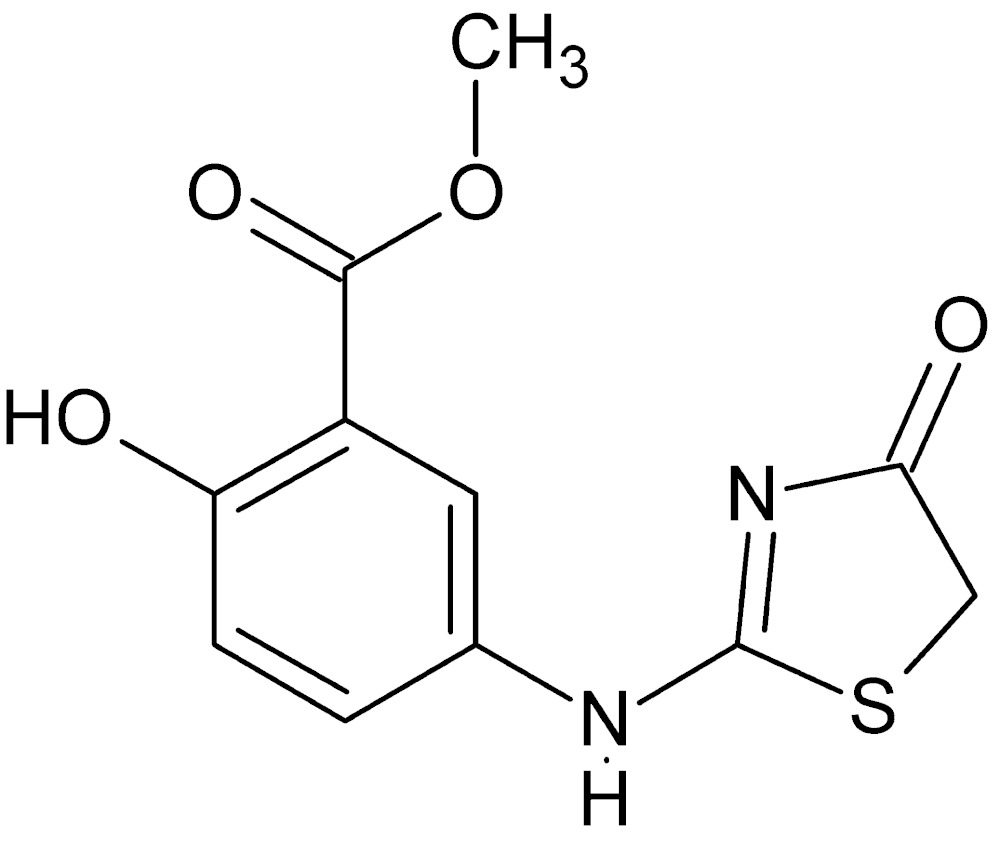


## Experimental   

### Crystal data  

C_11_H_10_N_2_O_4_S
*M* *_r_* = 266.27
Monoclinic, 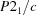

*a* = 4.7787 (1) Å
*b* = 25.4128 (7) Å
*c* = 18.9599 (5) Å
β = 90.841 (1)°
*V* = 2302.24 (10) Å^3^
*Z* = 8
Cu *K*α radiation
μ = 2.62 mm^−1^
*T* = 150 K
0.16 × 0.12 × 0.09 mm

### Data collection  

Bruker D8 VENTURE PHOTON 100 CMOS diffractometer
Absorption correction: multi-scan (*SADABS*; Bruker, 2014 ▸) *T*_min_ = 0.76, *T*_max_ = 0.80
17982 measured reflections
4582 independent reflections
3972 reflections with *I* > 2σ(*I*)
*R*_int_ = 0.031

### Refinement  

*R*[*F* ^2^ > 2σ(*F* ^2^)] = 0.034
*wR*(*F* ^2^) = 0.089
*S* = 1.04
4582 reflections
327 parameters
H-atom parameters constrained
Δρ_max_ = 0.29 e Å^−3^
Δρ_min_ = −0.26 e Å^−3^

### 

Data collection: *APEX2* (Bruker, 2014 ▸); cell refinement: *SAINT* (Bruker, 2014 ▸); data reduction: *SAINT*; program(s) used to solve structure: *SHELXT* (Sheldrick, 2015*a* ▸); program(s) used to refine structure: *SHELXL2014* (Sheldrick, 2015*b* ▸); molecular graphics: *DIAMOND* (Brandenburg & Putz, 2012 ▸); software used to prepare material for publication: *SHELXTL* (Sheldrick, 2008 ▸).

## Supplementary Material

Crystal structure: contains datablock(s) global, I. DOI: 10.1107/S2056989015006416/hg5436sup1.cif

Structure factors: contains datablock(s) I. DOI: 10.1107/S2056989015006416/hg5436Isup2.hkl

Supporting information file. DOI: 10.1107/S2056989015006416/hg5436Isup3.cml

**Fig. 1.**
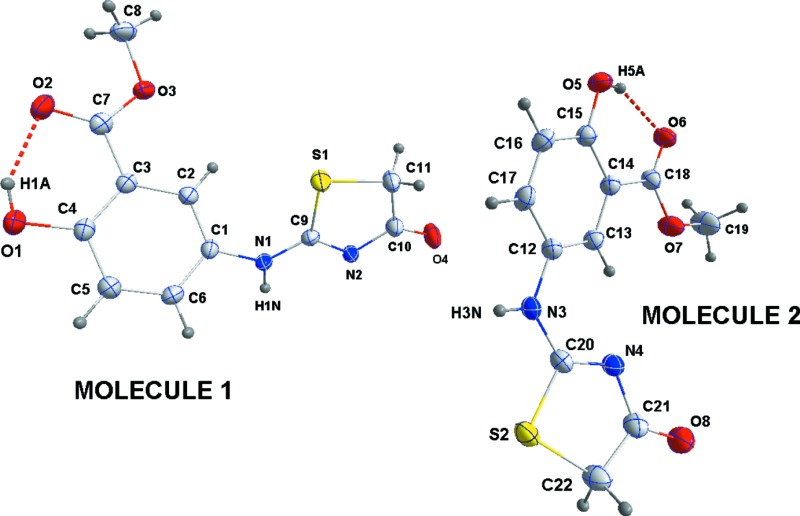
The asymmetric unit for the title compound with labeling scheme and 50% probability ellipsoids. The intramolecular hydrogen bonds are shown as dotted lines.

**Fig. 2.**
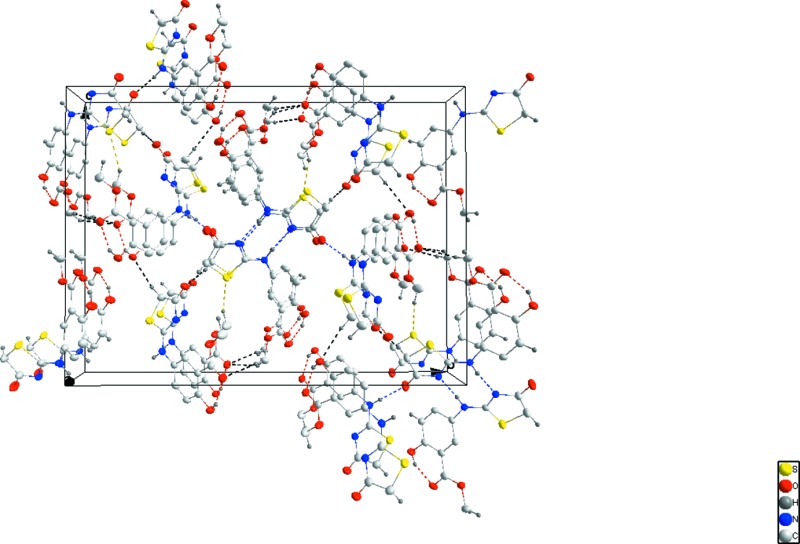
Packing viewed down the a axis with O—H···O (red) N—H···O (blue), N—H···N (blue), C—H···O (black) and C—H···S (yellow) interactions shown as dotted lines.

**Fig. 3.**
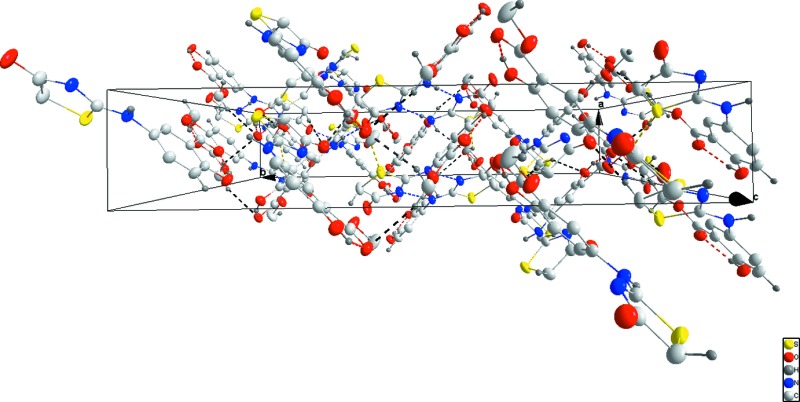
Packing viewed down the c axis. Key to dotted lines as for Figure 2.

. DOI: 10.1107/S2056989015006416/hg5436fig1.tif
The asymmetric unit for the title compound with labeling scheme and 50% probability ellipsoids. The intra­molecular hydrogen bonds are shown as dotted lines.

a . DOI: 10.1107/S2056989015006416/hg5436fig2.tif
Packing viewed down the *a* axis with O—H⋯O (red) N—H⋯O (blue), N—H⋯N (blue), C—H⋯O (black) and C—H⋯S (yellow) inter­actions shown as dotted lines.

c . DOI: 10.1107/S2056989015006416/hg5436fig3.tif
Packing viewed down the *c* axis. Key to dotted lines as for Fig. 2.

CCDC reference: 1056711

Additional supporting information:  crystallographic information; 3D view; checkCIF report

## Figures and Tables

**Table 1:** Hydrogen-bond geometry (, )

*D*H*A*	*D*H	H*A*	*D* *A*	*D*H*A*
O1H1*A*O2	0.84	1.87	2.6287(18)	150
O5H5*A*O6	0.84	1.92	2.6619(18)	147
N1H1*N*N2^i^	0.91	1.96	2.8624(19)	175
N3H3*N*O4^ii^	0.91	1.97	2.8703(18)	170
C11H11*A*O8^iii^	0.99	2.39	3.371(2)	171
C5H5S2^iv^	0.95	2.71	3.5043(18)	141

Symmetry codes: (i) 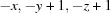; (ii) 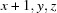; (iii) 
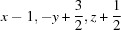
; (iv) 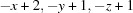.

## supplementary crystallographic information

### S1. Comment

5-Aminosalicylic acid (5-ASA) is a prototype drug that is commonly described for treatment of inflammatory bowel diseases (Abdel-Alim, *et al.*, 2005; Abdu-Allah, *et al.*, 2005). It was shown that 5-ASA has,also, chemopreventive and chemotherapeutic properties (Koelink, *et al.*, 2010). On the other hand 4-thiazolidinone derivatives have attracted continuing interest over the years because of their diverse biological activities, such as anti-inflammatory, anti-proliferative, antiviral, anticonvulsant, anti-diabetic, anti-hyperlipidemic, cardiovascular, anti-tubercular, antifungal, and antibacterial (Tripathi, *et al.*, 2014). Based in these findings, we were interested in the synthesis of hybrid molecules that combine both pharmacophores, therefore we report in this study the synthesis and crystal structure of the title compound.

The title compound contains two independent molecules in the asymmetric unit which differ primarily in the rotational orientation of the 5-membered, heterocyclic ring (Fig. 1). Each molecule contais a strong, intramolecular hydrogen bond (Table 1 and Fig. 1) which determines the orientation of the ester group. The molecules pack in a zigzag fashion (Fig. 3) assembled by intermolecular N—H···O, N—H···N, C—H···O and C—H···S interactions (Table 1 and Fig. 2)

### S2. Experimental

A solution of methyl 5-[(chloroacetyl)amino]-2-hydroxybenzoate (2.3 g, 9.5 mmol) and ammonium thiocyanate (1.5 g, 19.7 mmol) in 40 ml ethanol was refluxed for 3 h and allowed to stand overnight. The mixture was evaporated and the residue was washed with water and then recrystallized from ethanol/water to give the title compound (2.13 g, 85% yield); R­­_f_ = 0.25 (hexane:ethyl acetate, 2:1). Mp. 481–482 K.

### S3. Refinement

H-atoms attached to carbon were placed in calculated positions C—H = 0.95 - 0.98 Å) while those attached to nitrogen and oxygen were placed in locations derived from a difference map, refined initially to verify their presence and then their parameters adjusted to give N—H = 0.91 Å and O—H = 0.84 Å. All were included as riding contributions with isotropic displacement parameters 1.2 - 1.5 times those of the attached atoms.

### Crystal data

**Table:**

C_11_H_10_N_2_O_4_S	*F*(000) = 1104
*M**_r_* = 266.27	*D*_x_ = 1.536 Mg m^−^^3^
Monoclinic, *P*2_1_/*c*	Cu *K*α radiation, λ = 1.54178 Å
*a* = 4.7787 (1) Å	Cell parameters from 9975 reflections
*b* = 25.4128 (7) Å	θ = 4.2–74.5°
*c* = 18.9599 (5) Å	µ = 2.62 mm^−^^1^
β = 90.841 (1)°	*T* = 150 K
*V* = 2302.24 (10) Å^3^	Block, yellow-orange
*Z* = 8	0.16 × 0.12 × 0.09 mm

### Data collection

**Table:**

Bruker D8 VENTURE PHOTON 100 CMOS diffractometer	4582 independent reflections
Radiation source: INCOATEC IµS micro-focus source	3972 reflections with *I* > 2σ(*I*)
Mirror monochromator	*R*_int_ = 0.031
Detector resolution: 10.4167 pixels mm^-1^	θ_max_ = 74.5°, θ_min_ = 2.9°
ω scans	*h* = −5→5
Absorption correction: multi-scan (*SADABS*; Bruker, 2014)	*k* = −31→31
*T*_min_ = 0.76, *T*_max_ = 0.80	*l* = −21→23
17982 measured reflections	

### Refinement

**Table:**

Refinement on *F*^2^	Primary atom site location: structure-invariant direct methods
Least-squares matrix: full	Secondary atom site location: difference Fourier map
*R*[*F*^2^ > 2σ(*F*^2^)] = 0.034	Hydrogen site location: mixed
*wR*(*F*^2^) = 0.089	H-atom parameters constrained
*S* = 1.04	*w* = 1/[σ^2^(*F*_o_^2^) + (0.0435*P*)^2^ + 1.0566*P*] where *P* = (*F*_o_^2^ + 2*F*_c_^2^)/3
4582 reflections	(Δ/σ)_max_ = 0.001
327 parameters	Δρ_max_ = 0.29 e Å^−^^3^
0 restraints	Δρ_min_ = −0.26 e Å^−^^3^

### Special details

**Table:**

Geometry. All e.s.d.'s (except the e.s.d. in the dihedral angle between two l.s. planes)are estimated using the full covariance matrix. The cell e.s.d.'s are takeninto account individually in the estimation of e.s.d.'s in distances, anglesand torsion angles; correlations between e.s.d.'s in cell parameters are onlyused when they are defined by crystal symmetry. An approximate (isotropic)treatment of cell e.s.d.'s is used for estimating e.s.d.'s involving l.s. planes.
Refinement. Refinement of *F*^2^ against ALL reflections. The weighted *R*-factor *wR* andgoodness of fit *S* are based on *F*^2^, conventional *R*-factors *R* are basedon *F*, with *F* set to zero for negative *F*^2^. The threshold expression of*F*^2^ > σ(*F*^2^) is used only for calculating *R*-factors(gt) *etc*. and isnot relevant to the choice of reflections for refinement. *R*-factors basedon *F*^2^ are statistically about twice as large as those based on *F*, and *R*-factors based on ALL data will be even larger. H-atoms attached to carbonwere placed in calculated positions (C—H = 0.95 - 0.98 Å) while thoseattached to nitrogen and oxygen were placed in locations derived from adifference map, refined initially to verify their presence and then theirparameters adjusted to give N—H = 0.91 Å and O—H = 0.84 Å. Allwere included as riding contributions with isotropic displacementparameters 1.2 - 1.5 times those of the attached atoms.

### Fractional atomic coordinates and isotropic or equivalent isotropic displacement parameters (Å^2^)

**Table:**

	*x*	*y*	*z*	*U*_iso_*/*U*_eq_	
S1	0.20576 (11)	0.60092 (2)	0.64250 (2)	0.03308 (12)	
O1	0.8378 (3)	0.39435 (5)	0.78252 (7)	0.0340 (3)	
H1A	0.7774	0.4013	0.8228	0.041*	
O2	0.5442 (3)	0.43997 (5)	0.88146 (6)	0.0313 (3)	
O3	0.1993 (3)	0.49520 (5)	0.84894 (6)	0.0296 (3)	
O4	−0.2571 (3)	0.64326 (5)	0.48484 (7)	0.0460 (4)	
N1	0.1989 (3)	0.50385 (5)	0.58425 (7)	0.0244 (3)	
H1N	0.1522	0.4827	0.5471	0.029*	
N2	−0.0532 (3)	0.56801 (5)	0.52659 (7)	0.0263 (3)	
C1	0.3659 (4)	0.48031 (6)	0.63838 (8)	0.0234 (3)	
C2	0.3104 (3)	0.48582 (6)	0.70909 (8)	0.0235 (3)	
H2	0.1641	0.5085	0.7237	0.028*	
C3	0.4698 (3)	0.45798 (6)	0.75962 (8)	0.0224 (3)	
C4	0.6808 (4)	0.42367 (6)	0.73737 (9)	0.0253 (3)	
C5	0.7330 (4)	0.41851 (7)	0.66549 (9)	0.0295 (4)	
H5	0.8772	0.3957	0.6501	0.035*	
C6	0.5769 (4)	0.44630 (7)	0.61680 (9)	0.0264 (3)	
H6	0.6131	0.4423	0.5680	0.032*	
C7	0.4110 (4)	0.46286 (6)	0.83537 (9)	0.0244 (3)	
C8	0.1382 (4)	0.50350 (7)	0.92288 (9)	0.0341 (4)	
H8A	0.1074	0.4695	0.9458	0.051*	
H8B	−0.0304	0.5252	0.9269	0.051*	
H8C	0.2964	0.5215	0.9459	0.051*	
C9	0.1095 (4)	0.55291 (6)	0.58058 (8)	0.0235 (3)	
C10	−0.1141 (4)	0.62053 (7)	0.52919 (9)	0.0303 (4)	
C11	0.0118 (4)	0.64938 (7)	0.59232 (10)	0.0337 (4)	
H11A	−0.1376	0.6650	0.6213	0.040*	
H11B	0.1374	0.6779	0.5765	0.040*	
S2	0.79895 (10)	0.68556 (2)	0.30469 (2)	0.03288 (12)	
O5	−0.1163 (3)	0.86366 (5)	0.57459 (6)	0.0311 (3)	
H5A	−0.2396	0.8788	0.5500	0.037*	
O6	−0.3730 (3)	0.89682 (5)	0.45724 (7)	0.0315 (3)	
O7	−0.2248 (3)	0.86409 (5)	0.35416 (6)	0.0359 (3)	
O8	0.4930 (3)	0.78782 (5)	0.17658 (7)	0.0369 (3)	
N3	0.4978 (3)	0.72813 (5)	0.40544 (7)	0.0266 (3)	
H3N	0.5957	0.7034	0.4303	0.032*	
N4	0.4467 (3)	0.76550 (5)	0.29297 (7)	0.0273 (3)	
C12	0.3322 (3)	0.76357 (6)	0.44516 (9)	0.0244 (3)	
C13	0.1336 (3)	0.79618 (6)	0.41508 (9)	0.0242 (3)	
H13	0.1002	0.7952	0.3656	0.029*	
C14	−0.0191 (3)	0.83065 (6)	0.45723 (8)	0.0232 (3)	
C15	0.0258 (3)	0.83183 (6)	0.53042 (9)	0.0249 (3)	
C16	0.2281 (4)	0.79875 (7)	0.55986 (9)	0.0284 (4)	
H16	0.2617	0.7993	0.6094	0.034*	
C17	0.3800 (4)	0.76519 (7)	0.51822 (9)	0.0276 (4)	
H17	0.5181	0.7430	0.5391	0.033*	
C18	−0.2245 (4)	0.86683 (6)	0.42429 (9)	0.0261 (3)	
C19	−0.4239 (5)	0.89664 (8)	0.31670 (11)	0.0464 (5)	
H19A	−0.6106	0.8913	0.3361	0.070*	
H19B	−0.4260	0.8871	0.2666	0.070*	
H19C	−0.3704	0.9337	0.3219	0.070*	
C20	0.5553 (3)	0.73108 (6)	0.33708 (9)	0.0250 (3)	
C21	0.5600 (4)	0.76067 (7)	0.22716 (9)	0.0283 (4)	
C22	0.7842 (4)	0.71821 (7)	0.22087 (9)	0.0330 (4)	
H22A	0.9675	0.7342	0.2100	0.040*	
H22B	0.7338	0.6930	0.1829	0.040*	

### Atomic displacement parameters (Å^2^)

**Table:**

	*U*^11^	*U*^22^	*U*^33^	*U*^12^	*U*^13^	*U*^23^
S1	0.0490 (3)	0.0229 (2)	0.0269 (2)	0.00344 (17)	−0.01520 (19)	−0.00436 (15)
O1	0.0388 (7)	0.0389 (7)	0.0241 (6)	0.0151 (5)	−0.0018 (5)	0.0060 (5)
O2	0.0332 (7)	0.0394 (7)	0.0213 (6)	0.0014 (5)	−0.0020 (5)	0.0078 (5)
O3	0.0397 (7)	0.0296 (6)	0.0195 (6)	0.0054 (5)	0.0023 (5)	−0.0006 (5)
O4	0.0700 (10)	0.0286 (7)	0.0385 (8)	0.0144 (6)	−0.0250 (7)	0.0000 (6)
N1	0.0338 (8)	0.0220 (6)	0.0172 (6)	0.0042 (5)	−0.0053 (5)	−0.0024 (5)
N2	0.0373 (8)	0.0222 (6)	0.0193 (7)	0.0057 (6)	−0.0067 (6)	−0.0019 (5)
C1	0.0280 (9)	0.0206 (7)	0.0213 (8)	0.0010 (6)	−0.0042 (6)	0.0018 (6)
C2	0.0275 (9)	0.0209 (7)	0.0220 (8)	0.0015 (6)	−0.0007 (6)	−0.0007 (6)
C3	0.0254 (8)	0.0219 (7)	0.0200 (8)	−0.0018 (6)	−0.0013 (6)	0.0014 (6)
C4	0.0271 (9)	0.0251 (8)	0.0236 (8)	0.0019 (6)	−0.0029 (6)	0.0040 (6)
C5	0.0310 (9)	0.0315 (9)	0.0262 (9)	0.0087 (7)	0.0010 (7)	0.0005 (7)
C6	0.0317 (9)	0.0291 (8)	0.0185 (8)	0.0032 (7)	0.0010 (6)	−0.0006 (6)
C7	0.0271 (9)	0.0231 (7)	0.0231 (8)	−0.0051 (6)	−0.0005 (6)	0.0013 (6)
C8	0.0461 (11)	0.0347 (9)	0.0216 (9)	0.0007 (8)	0.0059 (8)	−0.0030 (7)
C9	0.0301 (9)	0.0228 (7)	0.0175 (7)	0.0012 (6)	−0.0018 (6)	−0.0013 (6)
C10	0.0413 (10)	0.0240 (8)	0.0253 (9)	0.0047 (7)	−0.0066 (7)	−0.0003 (7)
C11	0.0498 (12)	0.0216 (8)	0.0293 (9)	0.0050 (7)	−0.0116 (8)	−0.0010 (7)
S2	0.0378 (3)	0.0296 (2)	0.0314 (2)	0.01298 (17)	0.00235 (18)	0.00021 (17)
O5	0.0353 (7)	0.0344 (6)	0.0237 (6)	0.0065 (5)	0.0015 (5)	−0.0028 (5)
O6	0.0347 (7)	0.0282 (6)	0.0317 (7)	0.0084 (5)	0.0001 (5)	−0.0025 (5)
O7	0.0455 (8)	0.0370 (7)	0.0250 (6)	0.0180 (6)	−0.0089 (5)	−0.0021 (5)
O8	0.0466 (8)	0.0390 (7)	0.0250 (6)	0.0061 (6)	−0.0015 (5)	0.0053 (5)
N3	0.0309 (8)	0.0236 (7)	0.0253 (7)	0.0076 (6)	−0.0014 (6)	0.0039 (5)
N4	0.0316 (8)	0.0259 (7)	0.0242 (7)	0.0052 (6)	−0.0002 (6)	0.0011 (5)
C12	0.0265 (9)	0.0217 (7)	0.0250 (8)	0.0011 (6)	0.0018 (6)	0.0017 (6)
C13	0.0264 (9)	0.0244 (8)	0.0218 (8)	0.0001 (6)	−0.0022 (6)	0.0003 (6)
C14	0.0242 (8)	0.0220 (7)	0.0233 (8)	−0.0011 (6)	−0.0016 (6)	0.0008 (6)
C15	0.0262 (9)	0.0244 (8)	0.0241 (8)	−0.0024 (6)	0.0017 (6)	−0.0001 (6)
C16	0.0316 (9)	0.0330 (9)	0.0206 (8)	−0.0009 (7)	−0.0015 (7)	0.0027 (6)
C17	0.0280 (9)	0.0289 (8)	0.0257 (9)	0.0023 (7)	−0.0016 (7)	0.0070 (7)
C18	0.0288 (9)	0.0230 (8)	0.0265 (9)	0.0004 (6)	−0.0028 (7)	−0.0017 (6)
C19	0.0601 (14)	0.0448 (12)	0.0338 (11)	0.0248 (10)	−0.0156 (9)	0.0000 (8)
C20	0.0255 (8)	0.0212 (7)	0.0282 (9)	0.0025 (6)	−0.0003 (6)	−0.0017 (6)
C21	0.0317 (9)	0.0272 (8)	0.0260 (9)	−0.0015 (7)	−0.0026 (7)	−0.0026 (7)
C22	0.0344 (10)	0.0375 (10)	0.0272 (9)	0.0069 (8)	0.0002 (7)	−0.0032 (7)

### Geometric parameters (Å, º)

**Table:**

S1—C9	1.7500 (16)	S2—C20	1.7585 (16)
S1—C11	1.8043 (17)	S2—C22	1.7933 (19)
O1—C4	1.353 (2)	O5—C15	1.354 (2)
O1—H1A	0.8400	O5—H5A	0.8400
O2—C7	1.221 (2)	O6—C18	1.220 (2)
O3—C7	1.331 (2)	O7—C18	1.331 (2)
O3—C8	1.452 (2)	O7—C19	1.440 (2)
O4—C10	1.221 (2)	O8—C21	1.220 (2)
N1—C9	1.319 (2)	N3—C20	1.331 (2)
N1—C1	1.422 (2)	N3—C12	1.422 (2)
N1—H1N	0.9099	N3—H3N	0.9098
N2—C9	1.333 (2)	N4—C20	1.312 (2)
N2—C10	1.367 (2)	N4—C21	1.373 (2)
C1—C2	1.378 (2)	C12—C13	1.377 (2)
C1—C6	1.394 (2)	C12—C17	1.401 (2)
C2—C3	1.406 (2)	C13—C14	1.398 (2)
C2—H2	0.9500	C13—H13	0.9500
C3—C4	1.403 (2)	C14—C15	1.401 (2)
C3—C7	1.473 (2)	C14—C18	1.477 (2)
C4—C5	1.395 (2)	C15—C16	1.392 (2)
C5—C6	1.374 (2)	C16—C17	1.377 (2)
C5—H5	0.9500	C16—H16	0.9500
C6—H6	0.9500	C17—H17	0.9500
C8—H8A	0.9800	C19—H19A	0.9800
C8—H8B	0.9800	C19—H19B	0.9800
C8—H8C	0.9800	C19—H19C	0.9800
C10—C11	1.520 (2)	C21—C22	1.526 (2)
C11—H11A	0.9900	C22—H22A	0.9900
C11—H11B	0.9900	C22—H22B	0.9900
			
C9—S1—C11	89.67 (8)	C20—S2—C22	89.28 (8)
C4—O1—H1A	105.4	C15—O5—H5A	106.6
C7—O3—C8	116.14 (13)	C18—O7—C19	116.99 (14)
C9—N1—C1	127.82 (14)	C20—N3—C12	127.20 (14)
C9—N1—H1N	116.1	C20—N3—H3N	115.5
C1—N1—H1N	116.0	C12—N3—H3N	116.7
C9—N2—C10	112.07 (14)	C20—N4—C21	111.24 (14)
C2—C1—C6	119.94 (15)	C13—C12—C17	119.54 (15)
C2—C1—N1	123.06 (15)	C13—C12—N3	123.18 (15)
C6—C1—N1	116.70 (14)	C17—C12—N3	117.27 (14)
C1—C2—C3	120.12 (15)	C12—C13—C14	120.16 (15)
C1—C2—H2	119.9	C12—C13—H13	119.9
C3—C2—H2	119.9	C14—C13—H13	119.9
C4—C3—C2	119.52 (15)	C13—C14—C15	120.37 (15)
C4—C3—C7	119.59 (14)	C13—C14—C18	119.84 (15)
C2—C3—C7	120.85 (15)	C15—C14—C18	119.78 (15)
O1—C4—C5	117.43 (15)	O5—C15—C16	117.68 (15)
O1—C4—C3	123.12 (15)	O5—C15—C14	123.66 (15)
C5—C4—C3	119.44 (15)	C16—C15—C14	118.66 (15)
C6—C5—C4	120.33 (16)	C17—C16—C15	120.89 (16)
C6—C5—H5	119.8	C17—C16—H16	119.6
C4—C5—H5	119.8	C15—C16—H16	119.6
C5—C6—C1	120.63 (15)	C16—C17—C12	120.36 (16)
C5—C6—H6	119.7	C16—C17—H17	119.8
C1—C6—H6	119.7	C12—C17—H17	119.8
O2—C7—O3	123.05 (15)	O6—C18—O7	123.49 (15)
O2—C7—C3	123.57 (16)	O6—C18—C14	124.10 (15)
O3—C7—C3	113.38 (14)	O7—C18—C14	112.39 (14)
O3—C8—H8A	109.5	O7—C19—H19A	109.5
O3—C8—H8B	109.5	O7—C19—H19B	109.5
H8A—C8—H8B	109.5	H19A—C19—H19B	109.5
O3—C8—H8C	109.5	O7—C19—H19C	109.5
H8A—C8—H8C	109.5	H19A—C19—H19C	109.5
H8B—C8—H8C	109.5	H19B—C19—H19C	109.5
N1—C9—N2	119.84 (14)	N4—C20—N3	124.96 (15)
N1—C9—S1	122.80 (12)	N4—C20—S2	118.27 (13)
N2—C9—S1	117.33 (12)	N3—C20—S2	116.77 (12)
O4—C10—N2	123.68 (16)	O8—C21—N4	124.13 (17)
O4—C10—C11	121.66 (15)	O8—C21—C22	120.97 (16)
N2—C10—C11	114.66 (14)	N4—C21—C22	114.89 (15)
C10—C11—S1	106.27 (11)	C21—C22—S2	106.04 (12)
C10—C11—H11A	110.5	C21—C22—H22A	110.5
S1—C11—H11A	110.5	S2—C22—H22A	110.5
C10—C11—H11B	110.5	C21—C22—H22B	110.5
S1—C11—H11B	110.5	S2—C22—H22B	110.5
H11A—C11—H11B	108.7	H22A—C22—H22B	108.7
			
C9—N1—C1—C2	47.7 (3)	C20—N3—C12—C13	22.3 (3)
C9—N1—C1—C6	−138.67 (18)	C20—N3—C12—C17	−156.84 (17)
C6—C1—C2—C3	1.2 (2)	C17—C12—C13—C14	0.0 (2)
N1—C1—C2—C3	174.71 (15)	N3—C12—C13—C14	−179.07 (15)
C1—C2—C3—C4	−1.5 (2)	C12—C13—C14—C15	−0.9 (2)
C1—C2—C3—C7	−179.19 (15)	C12—C13—C14—C18	177.82 (15)
C2—C3—C4—O1	−178.04 (15)	C13—C14—C15—O5	−179.17 (15)
C7—C3—C4—O1	−0.3 (2)	C18—C14—C15—O5	2.1 (2)
C2—C3—C4—C5	1.3 (2)	C13—C14—C15—C16	1.1 (2)
C7—C3—C4—C5	178.98 (15)	C18—C14—C15—C16	−177.59 (15)
O1—C4—C5—C6	178.58 (16)	O5—C15—C16—C17	179.77 (15)
C3—C4—C5—C6	−0.8 (3)	C14—C15—C16—C17	−0.5 (3)
C4—C5—C6—C1	0.5 (3)	C15—C16—C17—C12	−0.4 (3)
C2—C1—C6—C5	−0.7 (3)	C13—C12—C17—C16	0.6 (3)
N1—C1—C6—C5	−174.61 (16)	N3—C12—C17—C16	179.74 (15)
C8—O3—C7—O2	1.9 (2)	C19—O7—C18—O6	−3.5 (3)
C8—O3—C7—C3	−177.46 (14)	C19—O7—C18—C14	178.15 (16)
C4—C3—C7—O2	3.0 (2)	C13—C14—C18—O6	177.39 (16)
C2—C3—C7—O2	−179.34 (16)	C15—C14—C18—O6	−3.9 (3)
C4—C3—C7—O3	−177.66 (14)	C13—C14—C18—O7	−4.2 (2)
C2—C3—C7—O3	0.0 (2)	C15—C14—C18—O7	174.46 (15)
C1—N1—C9—N2	−178.03 (16)	C21—N4—C20—N3	176.22 (16)
C1—N1—C9—S1	4.4 (3)	C21—N4—C20—S2	−3.0 (2)
C10—N2—C9—N1	−176.80 (16)	C12—N3—C20—N4	−6.2 (3)
C10—N2—C9—S1	0.9 (2)	C12—N3—C20—S2	172.97 (13)
C11—S1—C9—N1	176.98 (16)	C22—S2—C20—N4	4.75 (15)
C11—S1—C9—N2	−0.60 (15)	C22—S2—C20—N3	−174.50 (14)
C9—N2—C10—O4	179.11 (19)	C20—N4—C21—O8	179.46 (17)
C9—N2—C10—C11	−0.7 (2)	C20—N4—C21—C22	−1.1 (2)
O4—C10—C11—S1	−179.56 (17)	O8—C21—C22—S2	−176.26 (15)
N2—C10—C11—S1	0.3 (2)	N4—C21—C22—S2	4.23 (19)
C9—S1—C11—C10	0.17 (14)	C20—S2—C22—C21	−4.54 (13)

### Hydrogen-bond geometry (Å, º)

**Table:**

*D*—H···*A*	*D*—H	H···*A*	*D*···*A*	*D*—H···*A*
O1—H1*A*···O2	0.84	1.87	2.6287 (18)	150
O5—H5*A*···O6	0.84	1.92	2.6619 (18)	147
N1—H1*N*···N2^i^	0.91	1.96	2.8624 (19)	175
N3—H3*N*···O4^ii^	0.91	1.97	2.8703 (18)	170
C11—H11*A*···O8^iii^	0.99	2.39	3.371 (2)	171
C5—H5···S2^iv^	0.95	2.71	3.5043 (18)	141

Symmetry codes: (i) −*x*, −*y*+1, −*z*+1; (ii) *x*+1, *y*, *z*; (iii) *x*−1, −*y*+3/2, *z*+1/2; (iv) −*x*+2, −*y*+1, −*z*+1.
