# Supplementary figures and images for: Crystal structure of methyl 2-hy­droxy-5-[(4-oxo-4,5-di­hydro-1,3-thia­zol-2-yl)amino]benzoate
Source: Acta Crystallogr E Crystallogr Commun. 2015 Apr 9;71(Pt 5):o282–3. doi: 10.1107/S2056989015006416 (PMC4420109; doi:10.1107/S2056989015006416)

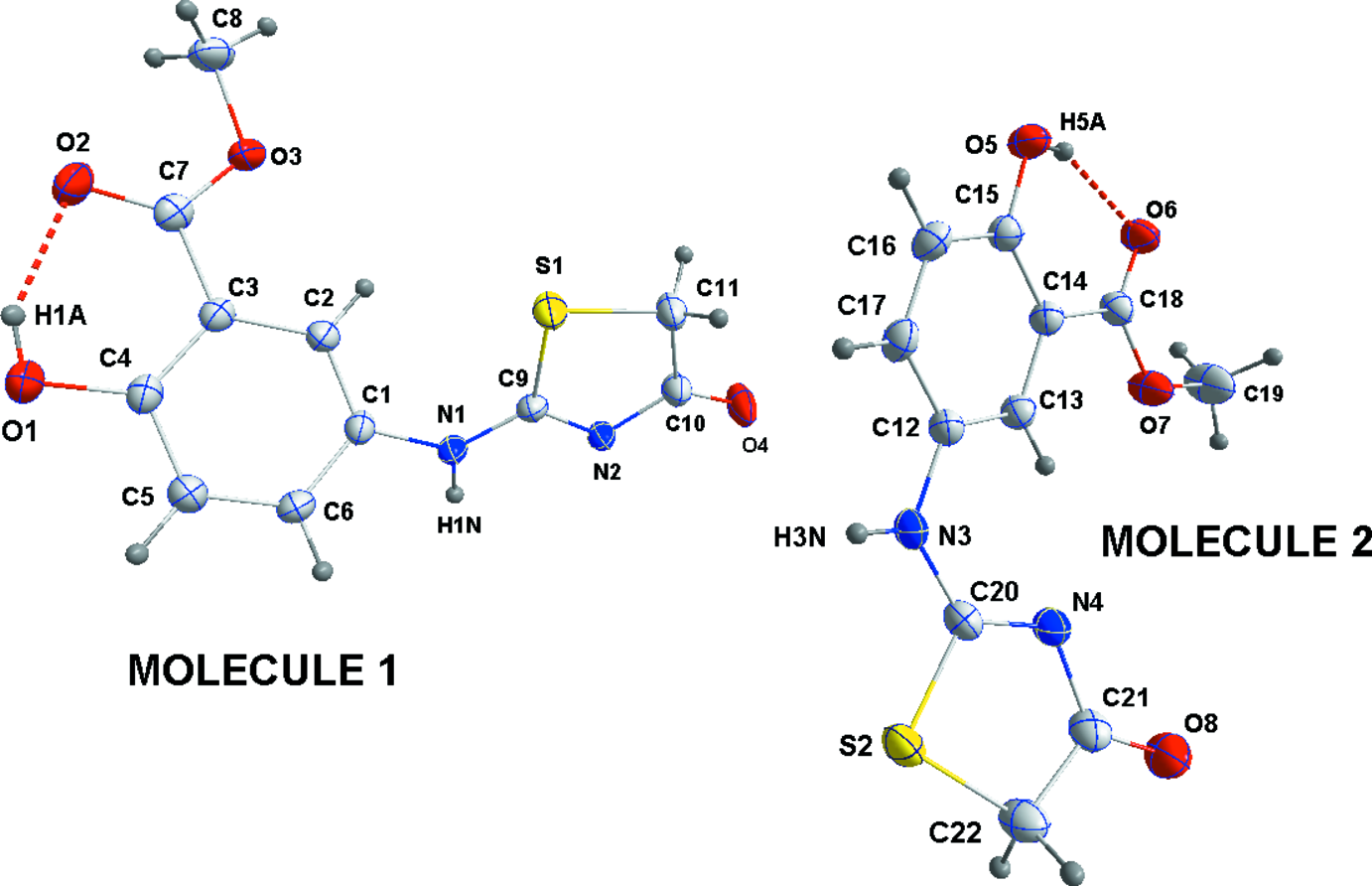

Supplement: Supplementary file 4 [file e-71-0o282-fig1.tif]

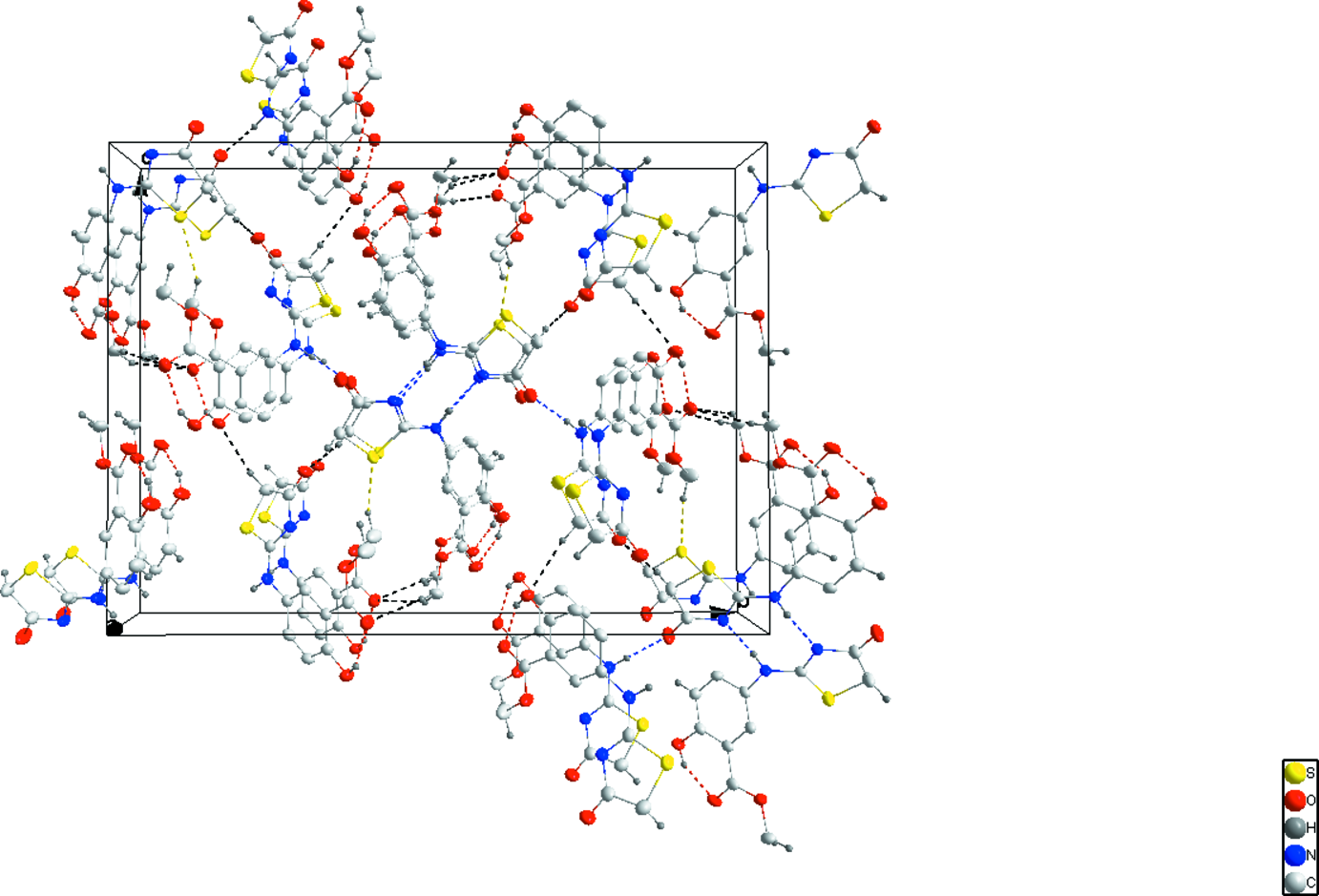

Supplement: Supplementary file 5 [file e-71-0o282-fig2.tif]

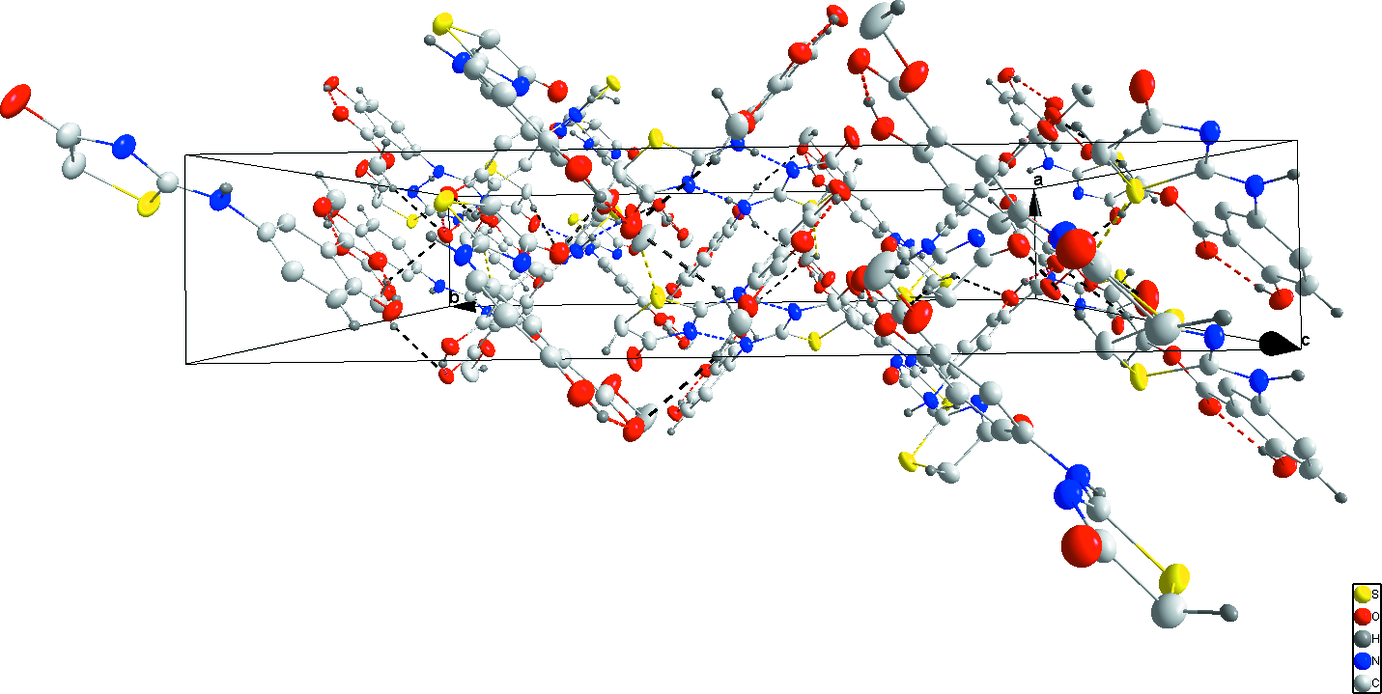

Supplement: Supplementary file 6 [file e-71-0o282-fig3.tif]
